# Supplementary material for: Admissions to paediatric medical wards with a primary mental health diagnosis: a systematic review of the literature
Source: Arch Dis Child. 2024 Feb 19;109(9):707–16. doi: 10.1136/archdischild-2023-326593 (PMC11347246; doi:10.1136/archdischild-2023-326593)
Supplement: Supplementary data [file archdischild-2023-326593supp002.pdf]

Appendix 2 Search strategy

From: 'Admissions to paediatric medical wards with a primary mental health diagnosis: a systematic review of the literature'

Search strategy
Search strategies (developed in conjunction with a clinical librarian) were tailored for each database. In summary, searches consisted of search terms combining three overall domains: (admissions) AND (paediatric or children's wards) AND (mental health diagnosis/problem).

Specific search terms were as follows: (admission* OR admitted OR admittance OR hospitalized OR hospitalised OR treated OR inpatient* OR in patient*) and (paediatric ward* OR children* ward* OR pediatric ward*) and (mental health* OR psychiatric or psychological).

PubMed
(("admission*"[All Fields] OR ("admit"[All Fields] OR "admits"[All Fields] OR "admitted"[All Fields] OR "admitting"[All Fields]) OR ("admittance"[All Fields] OR "admittances"[All Fields]) OR ("hospital s"[All Fields] OR "hospitalisation"[All Fields] OR "hospitalization"[MeSH Terms] OR "hospitalization"[All Fields] OR "hospitalised"[All Fields] OR "hospitalising"[All Fields] OR "hospitality"[All Fields] OR "hospitalisations"[All Fields] OR "hospitalizations"[All Fields] OR "hospitalize"[All Fields] OR "hospitalized"[All Fields] OR "hospitalizing"[All Fields] OR "hospitals"[MeSH Terms] OR "hospitals"[All Fields] OR "hospital"[All Fields]) OR ("therapy"[MeSH Subheading] OR "therapy"[All Fields] OR "treat"[All Fields] OR "treating"[All Fields] OR "treated"[All Fields] OR "treats"[All Fields])) AND ("paediatric wards"[All Fields] OR "children's wards"[All Fields] OR "pediatric wards"[All Fields]) AND ("children*"[All Fields] OR "young*"[All Fields] OR "adolescents*"[All Fields] OR "children and young people"[All Fields] OR "children and adolescents"[All Fields]) AND ("mental health*"[Author] OR "mental health diagnosis"[All Fields] OR "mental health problem"[All Fields] OR "mental health disorders"[All Fields] OR "mental health illness"[All Fields])) AND (1990/1/1:2023/4/24[pdat]).

Web of Science
ALL= ((admission* OR admitted OR admittance OR hospitalized OR treated) AND ("paediatric wards" OR "children's wards" OR "pediatric wards") AND (children* OR young* OR adolescents* OR "children and young people" OR "children and adolescents") AND (mental health* OR "mental health diagnosis" OR "mental health problem" OR "mental health disorders" OR "mental health illness")). Index data 1990-01-01 to 2023-04-24.

Embase
((admission* or admitted or admittance or hospitalized or hospitalised or treated or inpatient* or in patient*) and (paediatric ward* or children* ward* or pediatric ward*) and (mental health* or psychiatric or psychological)).mp. [mp=title, abstract, heading word, original title, keyword heading word, floating subheading word]. Limit to (full text and latest update and human and cochrane library and no language specified and yr="1990 - 2023").

PsycINFO
((admission* or admitted or admittance or hospitalized or hospitalised or treated or inpatient* or in patient*) and (paediatric ward* or children* ward* or pediatric ward*) and (mental health* or psychiatric or psychological)).mp. [mp=title, abstract, heading word, table of contents, key concepts, original title, tests & measures, mesh word]. Limit to (full text and apa psycarticles journals and all journals and latest update and human and yr="1990 - 2023" and open access).

Google Scholar
(admissions) AND (paediatric OR pediatric wards) AND (mental health).
