# Supplementary material for: Admissions to paediatric medical wards with a primary mental health diagnosis: a systematic review of the literature
Source: Arch Dis Child. 2024 Feb 19;109(9):707–16. doi: 10.1136/archdischild-2023-326593 (PMC11347246; doi:10.1136/archdischild-2023-326593)
Supplement: Supplementary data [file archdischild-2023-326593supp003.pdf]

Table S1. CASP Qualitative Studies Checklist*

		Criteria for qualitative studies										
#	Authors	1	2	3	4	5	6	7	8	9	10	Assessment
1	Chang et al (2023)	Y	Y	Y	Y	Y	C	Y	Y	Y	Y	High
2	Wu and Chen (2021)	Y	Y	Y	Y	Y	C	Y	Y	Y	Y	High
3	Worsley (2019)	Y	Y	N	Y	Y	N	C	Y	Y	Y	High
4	Hampton et al (2015)	Y	Y	Y	Y	Y	Y	Y	Y	Y	Y	High
5	Ramjan and Gil (2012)	Y	Y	C	Y	Y	N	Y	C	Y	C	Moderate
6	Happell et al (2009)	Y	Y	Y	Y	Y	C	Y	N	Y	Y	High
7	Watson (2006)	Y	Y	N	N	Y	N	N	C	Y	C	Low
8	Anderson et al (2003)	Y	Y	Y	C	Y	N	C	Y	Y	Y	High
9	King and Turner (2000)	Y	Y	Y	C	Y	N	Y	C	Y	C	Moderate

*CASP criteria for qualitative studies: 1. Was there a clear statement of the aims of the research?; 2. Was a qualitative methodology appropriate?; 3. Was the research design appropriate to address the aims of the research?; 4. Was the recruitment strategy appropriate to the aims of the research?; 5. Was the data collected in a way that addressed the research issue?; 6. Has the relationship between researcher and participants been adequately considered?; 7 Have ethical issues been considered?; 8. Was the data analysis sufficiently rigorous?; 9. Is there a clear statement of the findings?; 10. How valuable is the research? (Y: Yes, N: No, C: Can't tell)

Table S2. AXIS scale for cross-sectional studies*

		Kölch et al (2023)	Ibeziako et al (2022)	Duarte and Zelaya (2019)	Plemmons et al (2018)	Wallis (2018)	Gallagher et al (2017)	Santillanes et al (2017)	Claudius et al (2014)	Case et al (2007)	Levine et al (2005)	Smith et al (2014)	Mansbach et al (2003)	Ramritu et al (2002)	Valdivia et al (2001)	Gasquet and Choquet (1994)
Introduction	Were the aims/objectives of the study clear?	Y	Y	Y	Y	Y	Y	Y	Y	Y	Y	Y	Y	Y	Y	Y
Methods	Was the study design appropriate for the stated aim(s)?	Y	Y	Y	Y	Y	Y	Y	Y	Y	Y	Y	Y	Y	Y	Y
	Was the sample size justified?	N	N	Y	Y	N	N	N	N	Y	Y	N	N	N	N	N
	Was the target/reference population clearly defined? (Is it clear who the research was about?)	N	Y	Y	Y	Y	Y	Y	Y	Y	Y	Y	Y	Y	Y	Y
	Was the sample frame taken from an appropriate population base so that it closely represented the target/reference population under investigation?	Y	Y	Y	Y	Y	Y	Y	Y	Y	Y	Y	Y	Y	Y	Y
	Was the selection process likely to select subjects/participants that were representative of the target/reference population under investigation?	Y	Y	Y	Y	Y	Y	Y	Y	Y	Y	Y	Y	Y	Y	Y
	Were measures undertaken to address and categorize non-responders?	N	N	N	N	N	N	N	N	N	N	N	Y	N	N	N
	Were the risk factor and outcome variables measured appropriate to the aims of the study?	Y	Y	Y	Y	Y	Y	Y	Y	Y	Y	Y	Y	Y	Y	Y
	Were the risk factor and outcome variables measured correctly using instruments/measurements that had been trialled, piloted or published previously?	Y	Y	Y	Y	Y	Y	Y	Y	Y	Y	Y	Y	Y	Y	Y
	Is it clear what was used to determined statistical significance and/or precision estimates? (e.g., p values, CIs)	Y	Y	NA	Y	N	N	Y	N	Y	Y	N	Y	NA	NA	N
	Were the methods (including statistical methods) sufficiently described to enable them to be repeated?	Y	Y	Y	Y	N	Y	Y	Y	Y	Y	N	N	N	N	Y
Results	Were the basic data adequately described?	Y	Y	Y	Y	Y	Y	Y	Y	Y	Y	Y	Y	Y	Y	Y
	Does the response rate raise concerns about non-response bias?	ND	ND	ND	ND	ND	ND	ND	ND	ND	ND	ND	N	ND	N	N
	If appropriate, was information about no responders described?	N	N	N	N	N	N	N	N	N	N	N	Y	N	N	N
	Were the results internally consistent?	Y	Y	Y	Y	Y	Y	Y	Y	Y	Y	Y	Y	Y	Y	Y
	Were the results for the analyses described in the methods, presented?	N	Y	Y	Y	N	Y	Y	Y	Y	Y	Y	Y	Y	Y	Y
Discussion	Were the authors' discussions and conclusions justified by the results?	Y	Y	Y	Y	N	Y	Y	Y	Y	Y	Y	Y	Y	Y	Y
	Were the limitations of the study discussed?	Y	Y	Y	Y	N	Y	Y	Y	Y	Y	N	Y	N	N	N
Others	Were there any funding sources or conflicts of interest that may affect the authors' interpretation of the results?	NDis	N	NDis	N	NDis	N	N	N	NDis	NDis	NDis	NDis	NDis	NDis	NDis
	Was ethical approval or consent of participants attained?	NS	Y	Y	Y	NS	Y	Y	Y	NS	Y	Y	Y	Y	NS	NS

*The tool does not provide a numerical scale for assessing the quality of the study, it has areas to record assessment using "Yes", "No" or "Don't Know/comments" answer for each of the 20 questions.
Abbreviations: Y: Yes; N: No; DK: Don't know; Comments: not described (ND), not disclosed (NDis), not stated (NS), not applicable (NA)
